# The effect of storage delay and storage temperature on orthopaedic surgical samples contaminated by *Staphylococcus Epidermidis*

**DOI:** 10.1371/journal.pone.0192048

**Published:** 2018-03-19

**Authors:** Maïté Van Cauter, Olivier Cornu, Jean-Cyr Yombi, Hector Rodriguez-Villalobos, Ludovic Kaminski

**Affiliations:** 1 Department of Orthopedic Surgery, Cliniques Universitaires Saint Luc, Brussels, Belgium; 2 Department of Internal Medicine and Infectious Diseases, Cliniques Universitaires Saint Luc, Brussels, Belgium; 3 Department of Microbiology, Cliniques Universitaires Saint Luc, Brussels, Belgium; Universidade Nova de Lisboa Instituto de Higiene e Medicina Tropical, PORTUGAL

## Abstract

**Background:**

Prosthetic Joint Infection (PJI) is a rare but devastating complications with high morbitity and mortality. The identification of the causal microorganism remains crucial and determines therapeutic strategies and success. Microbiology cultures remain the common method to diagnose PJI. Unfortunately, 14% of intra-articular punctures remain negative after culture. The microorganisms are best detected by inoculation of microbiology samples in blood culture bottles (Bactec), or after sonication of the implant and polymerase chain reaction (PCR). The identification of the causal microorganism remains crucial and determines therapeutic success.

**Objectives:**

This study was conducted to assess the effect of culture lead time and sample storage temperature on the detection of the pathogen.

**Methods:**

We obtained bone fragments from femoral heads during primary arthroplasty. Bone fragments were contaminated with a strain of *Staphylococcus epidermidis*. Four set-ups with different combinations of storage delay and storage temperature were tested.

**Results:**

Our study shows the need to cultivate as soon as possible and optimally within 2h after the completion of sampling. Temporary storage in a refrigerator at 4°C also appears to have a positive influence on bacterial viability. At present, these conclusions concern only the *Staphylococcus Epidermidis*. Others studies are requested to generalize this conclusion to other bacteria.

## Introduction

The rise in the number of joint replacement procedures performed results in an increasing number of complications. The most severe complications include Periprosthetic Joint Infection (PJI). PJIs occur in 1–4% of patients after primary total hip or knee replacements and 3.2–7% of patients after revision arthroplasties [1]. The management of PJI remains difficult, time consuming and expensive. The identification of the microorganism responsible for the infection is of great importance [2]. This identification can be done in multiple ways: standard cultures on a Petri dish, culture vials type Bactec blood culture (Becton-Dickinson, USA), Polymerase Chain Reaction (PCR) analysis, sonication of the implant and development culturing the inoculum with embodiment in addition to a facultative PCR [3]. The microorganism should preferably be identified preoperatively from joint aspiration using cultures samples or PCR methods to optimize the antibiotherapy. However, this puncture remains negative in 14% of cases (3) because the amount of liquid removed is insufficient or because the culture is negative or false negative. The main reason from false negative cultures is the use of antibiotics before the culture. Another reason is that some bacteria are covered with a biofilm, some are slow-growing (dormant bacteria) and thus less easily cultivated [2], [4–6]. Sonication was developed to reduce this phenomenon and finally to improve the detection of the microorganism during PIJ.

In our institution, fluid samples are poured into blood culture bottles (Bactec Becton Dickinson, USA) to potentiate bacterial growth. The tissue samples are sent to the laboratory in sterile jars for microbiology and histology analysis. Correct sampling, transfer condition (temperature), and duration are essential in conducting such an appropriate process.

In our daily practice, the samples reach the microbiology laboratory with a very wide range of delay and are stored at 4°C for another varying duration until sowing (ranging from 2h to 24h). However, the optimal delay before culture and storage temperature to properly identify micro-organisms remains unknown. Therefore, our research investigates the effect of time between the surgical removal and sowing of tissue samples as well as the influence of storage temperature on bacterial growth. We focused this research on the *Staphylococcus epidermidis* who is one of the most comment microorganism responsible of PJI. 39.3% of joint infection in Europe and 20.2% in United States[7].

## Materials and methods

The bone material was been obtained from different femoral heads removed during primary hip prostheses surgery. Therefore, this bone was not previously infected.

The femoral heads were intersected in sterile conditions of about 125mm^3^ fragment. The fragments were stored in the freezer before contamination with *Staphylococcus Epidermidis*. The contamination was carried out under sterile conditions in order to avoid external contamination during handling. The various manipulations performed are summarized as follows: A strain of *Staphylococcus epidermidis* isolated from a chronically infected patient, bearer of a hip joint prosthesis was cultured on Petri-dish, we contaminated a saline solution with this strain to obtain a solution at a concentration of 0.5 McFarland, which allows obtaining reproducible turbidity. Various dilutions were performed to obtain a final contamination concentration of 1.5x10^4^cells per mL. A second 200 mL solution of sterile physiological saline was also prepared for the negative control group. Both solutions were left at 4°C for 30 min to bring the bacteria into the sessile phase. Then we added the bone fragments to these solutions. The solution was incubated for 30 min. The incubator was set at 5°C and at 5% CO_2_ and 140 RPM (revolutions per minute).

The bone fragments were separated from the solution by filtration and placed in sterile jars. Several experiments were performed according to the sample storage temperature and the culturing period. The samples were stored either in a refrigerator at 4°C or at room temperature (20 +-2°C). Sowing on agar Petri dishes (Columbia agar with 5% sheep blood) was done after specific times, ranging from time 0 to 24 h. For each experiment and each delay of sowing, a bone fragments from the negative control group was seeded on a Petri dish ranging for a total of 50 control samples. Each Petri dish was numbered and the microbiologist conducted the counting at 24h and 48h post sowing as single blind. In case two different types of colonies grew, it was also scored. The bacteriologist was not aware of the negative control groups.

### 1st experiment

Of 90 bone fragments collected, 55 were kept at room temperature during the entire experiment while the other 35 were kept at room temperature for the first 2h, which is the average time to route the sample from the operating room to the laboratory. After 2h, the samples were then placed in the refrigerator at 4°C. Sowing on Petri dish was performed on both bone groups at different times: T0, T30min, T1h, T2h, T4h, T6h, T8h, T12h, T16h, T24h and T48h. At each time, 5 bone fragments were plated on 5 different boxes. T0 corresponds to a sample taken in the operating room by the surgeon and immediately put on Petri dish. The objective of this experiment was to determine if the routing time and temperature influenced the growth and the interest of realizing the sowing immediately in the operating room.

### 2nd experiment

Of 45 bone fragments, 25 fragments were stored at room temperature and sowed at times T0, T30min, T1h, T2h and T4h. 20 more bone fragments were placed in the refrigerator at T0 and sown at T30min, T1h, T2h and T4h. This set-up would represent a situation where the fragment is sown by the surgeon during the intervention or immediately after (T0-T4h).

### 3rd experiment

The 3^rd^ experiment set-up was applied on 45 previously autoclaved bone fragments. 25 bone fragments were stored at room temperature and collected and seeded onto Petri dishes at times T0, T30min, T1h, T2h and T4h. 20 bone fragments were placed in the refrigerator at T0 and sown at T30 min, T1h, T2h and T4h. Since all patients received antibioprophylaxis (2g Kefzol, Teva Pharma AG, Basel) half an hour prior to surgery, and that the bone also contained the patient's antibodies, which might have caused a bacteriostatic or bactericidal effect on bacteria, the bone was autoclaved in order to assess the effects of the antibiotics or antibodies.

### 4th experiment

The contaminating solution was diluted to a greater extent in order to obtain a concentrated solution of 7.5 x10^2^ bacteria per mL. 25 bone fragments stored at room temperature were investigated at times T0, T30min, T1h, T2h and T4h.

Experiment 1 and 2 were jointly analyzed as they relate to the same concentration and the same conditions. Experiment 3 (bones autoclaving) and experiment 4 (low concentration of bacteria) were analyzed separately.

#### Statistical analysis

To explore the data, we performed a univariate and bivariate analysis, and then focused on inference and correlation. For continuous variables, their normal distribution was tested using a Kolmogorov-Smirnov test and consequently presented in terms of median (25^th^-75^th^ percentile) as the normality could not be assumed (i.e. non Gaussian distribution of continuous variables). Accordingly, we performed non-parametric tests: Mann Whitney U test was used for inference while Spearman Rho was used for correlations. All data were bilaterally tested with a level of significance of 0.05. All statistics were performed using SPSS software (v.20, SPSS, Inc., Chicago, IL, USA)

Ethic statement: In our institution we collected femoral heads for our Human tissue Bank. All patient signed a written consent. Our study received also the agreement of the ethics committee of our institution (Comité Ethique Facultaire de l’Université Catholique de Louvain No. B403201317725).

## Results

**Fig 1:**
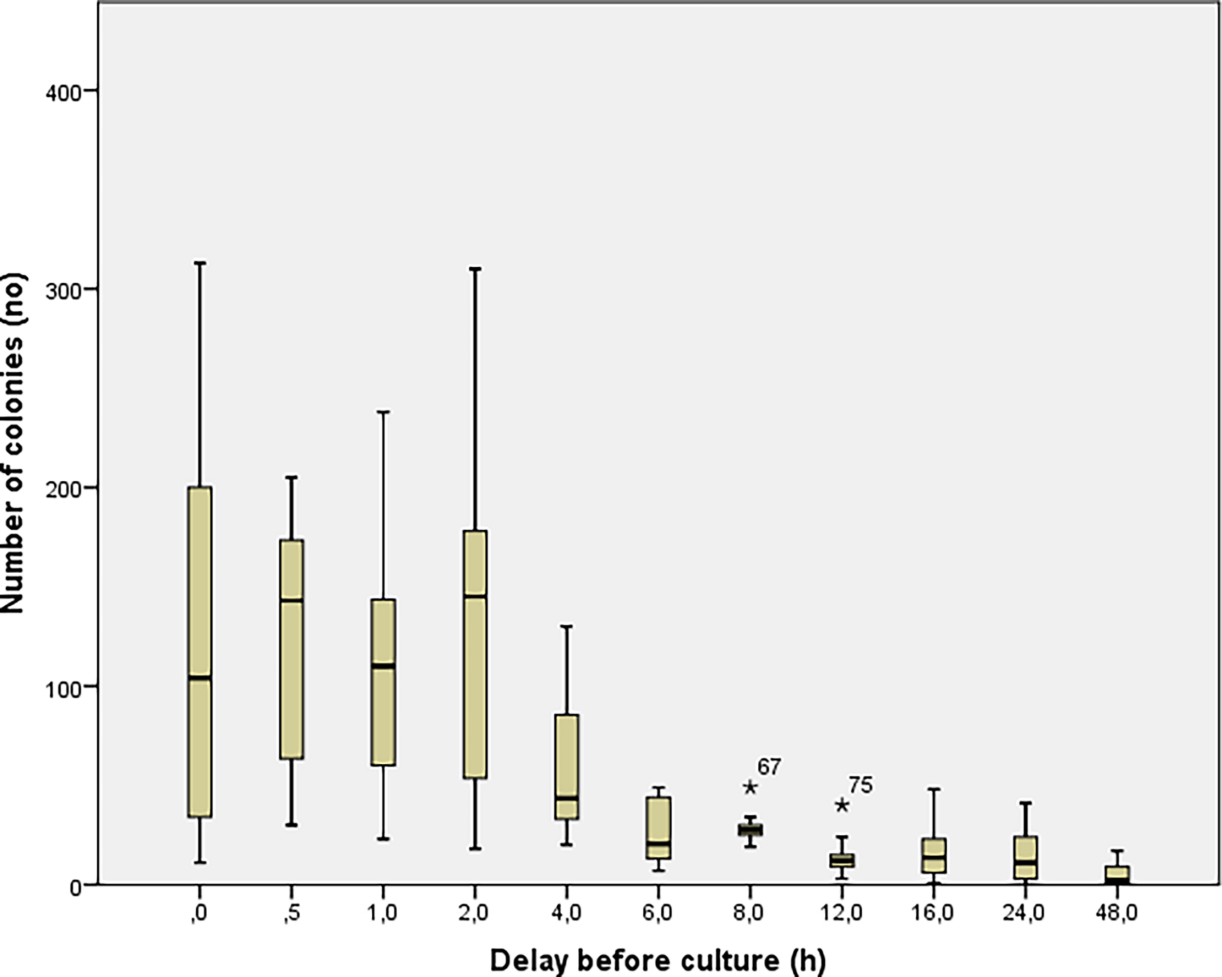
Results from experiments 1 and 2. The graph shows the bacterial growth according to the delay before sowing.

**Fig 2:**
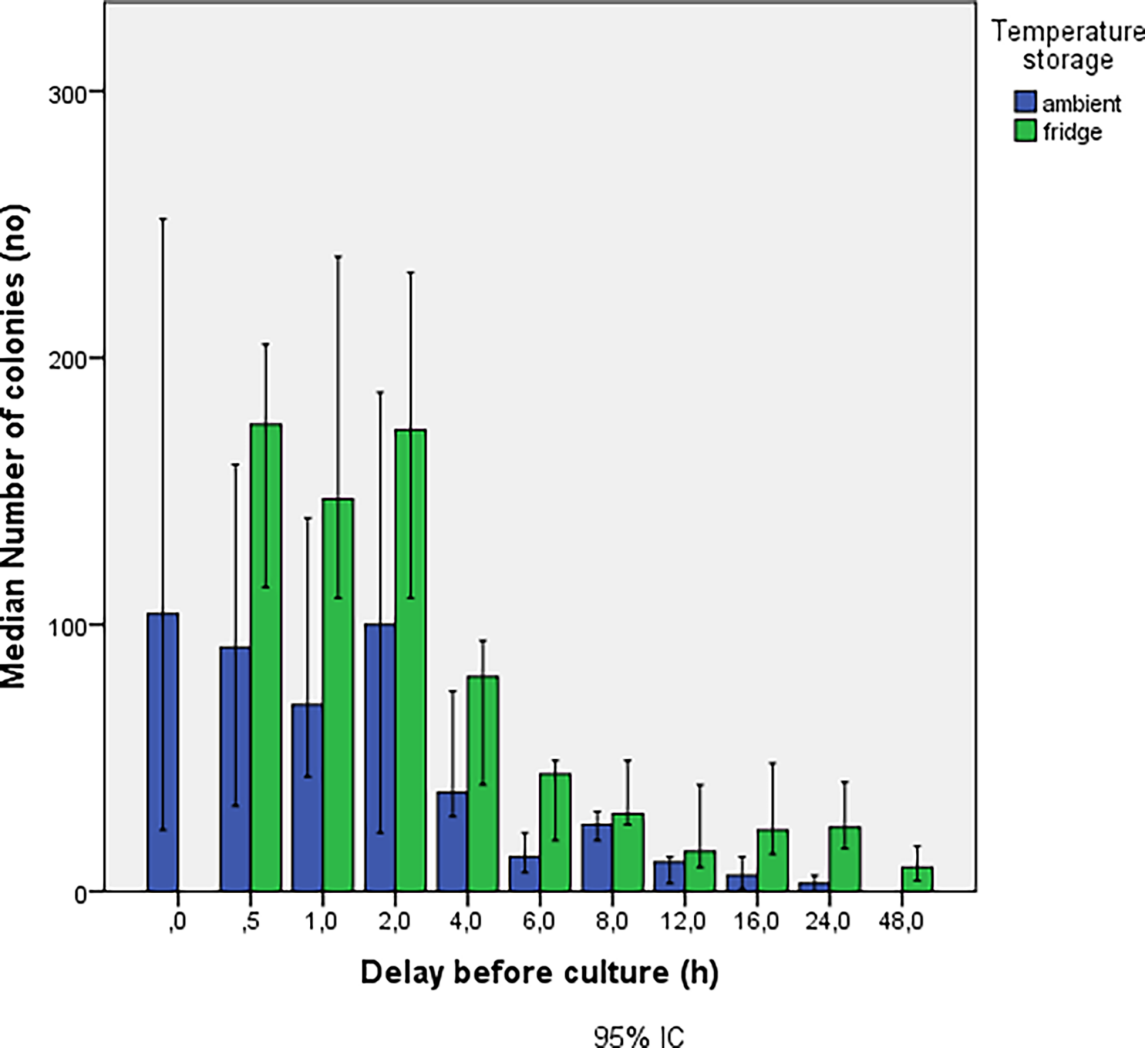
Results from experiments 1 and 2. In function of the storage temperature.

**Fig 3:**
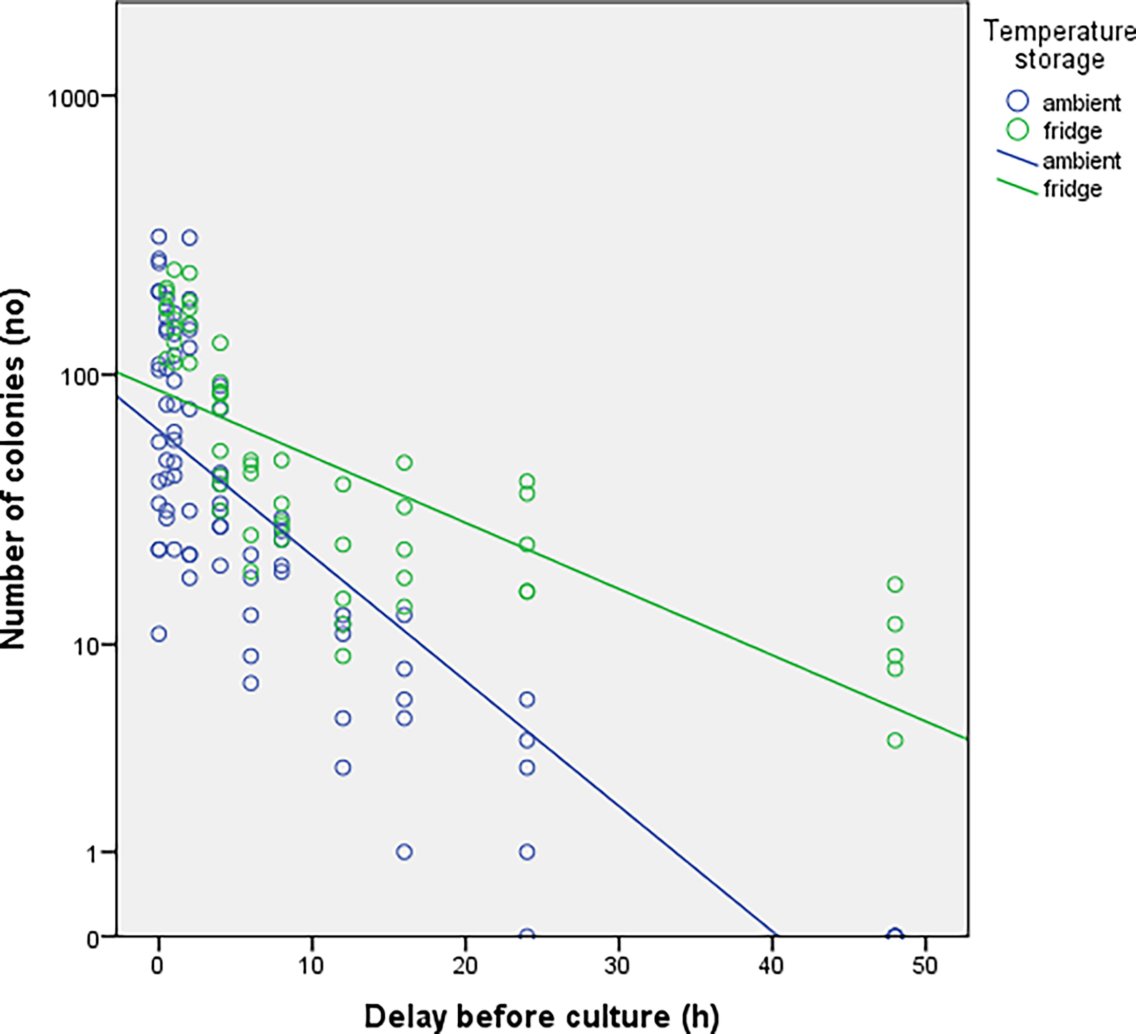
Results from experiments 1 and 2. The graph show the trend of the growing in function of the delay before sowing at to different temperature storage.

**Fig 4:**
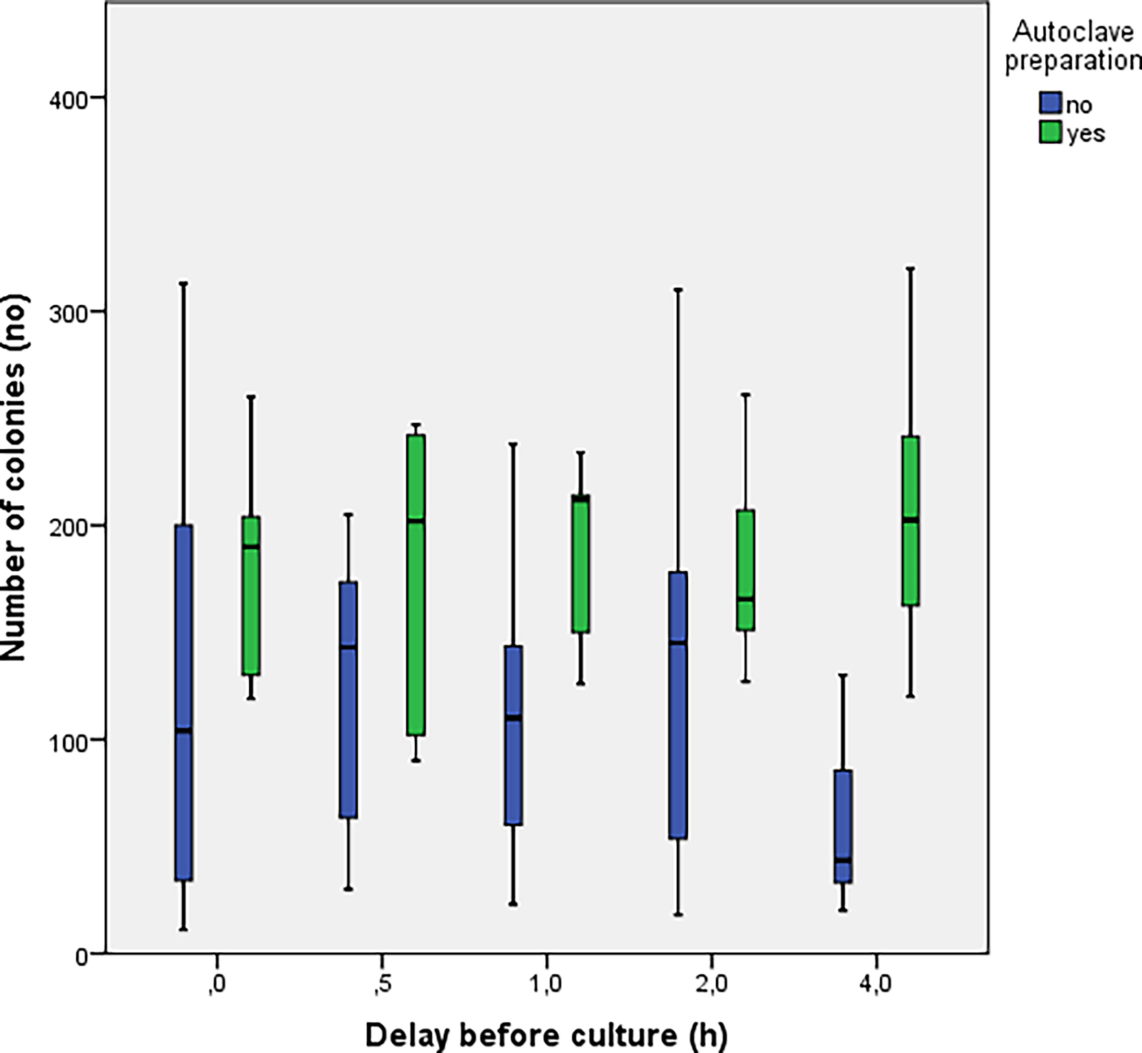
Results for the 3rd experiment. In green the bone preparation with autoclave and in blue without autoclave.

The number of colonies obtained based on the delay before sowing is shown in Fig 1 where results from experiments 1 and 2 were merged. Irrespective of the storage temperature, a decrease in the number of colonies was observed for delays before culturing of 2h with a trend towards inexistent crops at time 48h. In Fig 2, the groups at room temperature (blue) and at 4°C (green) from experiment 1 and 2 were split in order to determine the potential influence of temperature on bacterial growth. The concentration of bacteria was consistently higher in the group stored at 4°C at each delay before culturing. Two distinct trends can be seen as a function of storage of samples at room temperature compared to those stored in the refrigerator (Fig 3). The number of colonies decays much faster when samples are stored at room temperature. In experiment 3, the number of colonies observed from autoclaved fragments was significantly higher (p<0.05) at each delay of culturing, compared to those obtained from a bone fragment not autoclaved (Fig 4).

**Fig 5:**
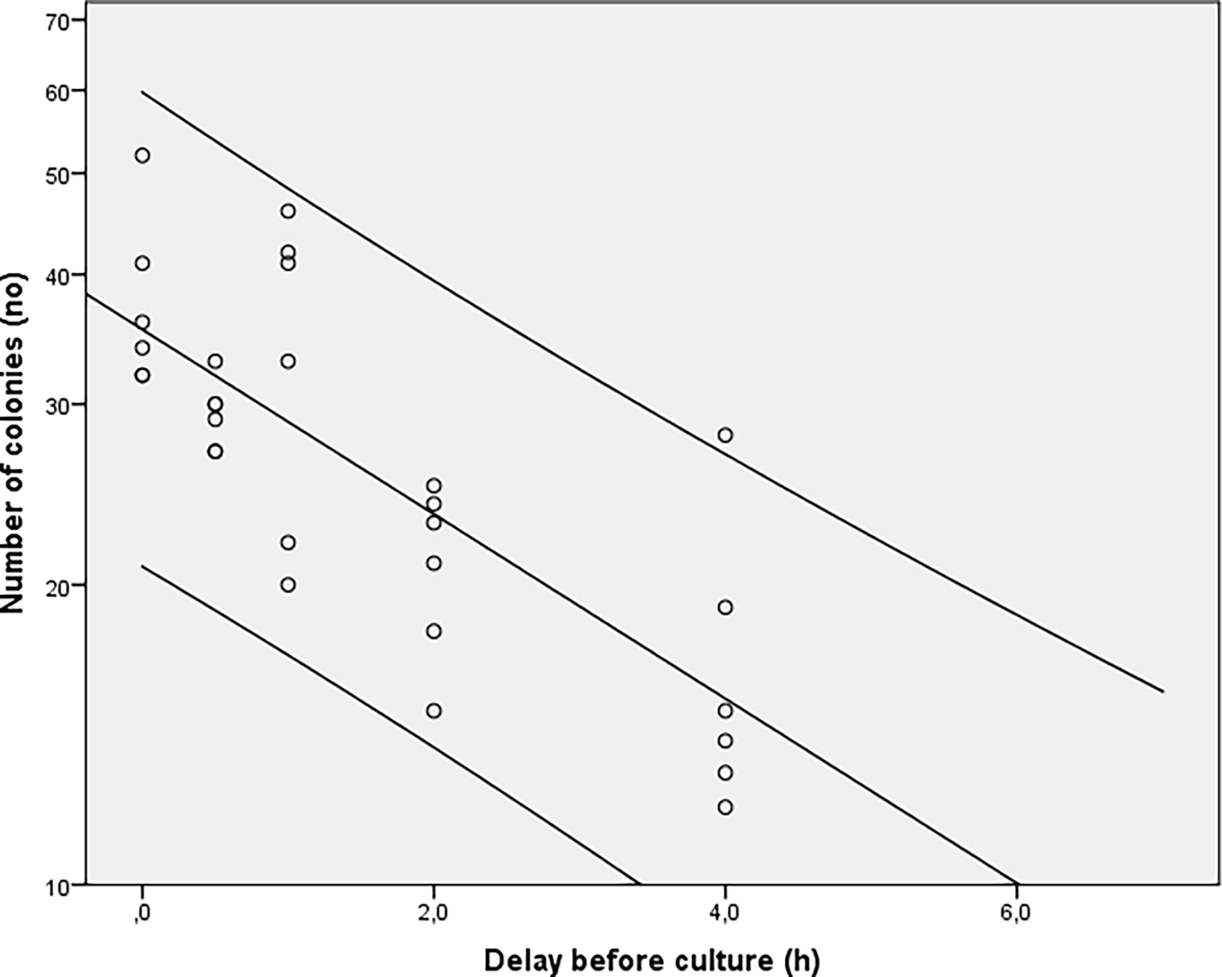
Results for the 4th experiment. Number of colonies tends to completely disappear when delay before sowing is longer than 6h.

In Experiment 4, where the *Staphylococcus epidermidis* concentration was reduced to 7.5 x 10^2^ cells per mL, the observed median number of colonies was lower than 20 after 4h. The aim of this experiment was to mimic *in vivo* conditions with bacteria in sleeping phase (slow growing). This suggests that the number of colonies tends to completely disappear when delay before sowing is longer than 6h (Fig 5). Readings from the control group (N = 50) resulted in two false positive contaminations due to handling, *i*.*e*. 4% of all controls.

## Discussion

The most important finding of our study is that the number of colonies cultured from a PJI is higher when samples are stored in a refrigerator before sowing, when delay before culture is shorter than 2 hours, and that PJI in vivo conditions are less likely identifiable when delay before culture exceeds 4 hours. Laboratory manipulations are a source of 4% false positive results in case of staphylococcus epidermidis.

In the event of a PJI, a proper identification of the infectious microorganism is essential to adequately determine the antibiotic therapy and optimize the surgery efficiency. The assessment of the strength of the pathogen may also influence the surgical strategy. A failure to identify surgery-induced germs in its early stage leads to heavy and expensive treatments with a risk of toxicity, which might induce their own set of complications. Conversely, generic treatment use in case of unknown microorganism often leads to poor medico-surgical management strategies, ultimately resulting in great failure. The cause of joint infections varies and can be due to different germs [8]. 65% of PJI originates from Gram-positive cocci like *Staphylococcus Aureus*, coagulase negative staphylococci(CNS) or *Streptococcus spp*. Polymicrobial infections account for 20%, gram negative *Bacilli* for 7%. The germs of the last 7% remain unknown, making the elaboration of an appropriate treatment difficult. The identification are make more difficult in case of slow growing micro-organism such as CNS. Coagulase negative is responsible for 20 to 39% of PJI.

The treatment of a chronic prosthetic joint infection usually proceeds in two distinct steps: first the infected material is removed and a spacer with antibiotics is applied. Secondly, a new prosthesis is placed after a delay of 4 to 6 weeks. During the first step, two situations might arise; either the germ of the infection is identified and specific antibiotics can be administered, or the puncture failed to identify the germ and empiric antibiotic applies in that instance. In the latter case, the determination of the causal agent is required in order to adapt and reduce the spectrum of antibiotics. The cultures resulting from the first step either provide positive result and allows for an adequate treatment, or provide negative results, calling for additional action. Cessation of the empirical antibiotic treatment during a therapeutic window of minimum 14 days should allow taking a new sample for additional analysis. This was proposed by Zimmerli [5], [6], [9] who removed the infected material as a first stage but placed the spacer only when the germ was identified.

To our knowledge, few studies have demonstrated the influence of conditions before sowing. Chronic infections are known as being one particular challenge due to the biofilm. In this condition, growth of bacteria is suboptimum. Our study demonstrates the need to optimize the mode of temporary storage before sowing. We are aware that it is not always practicable for the laboratory to make the sowing as fast as possible. According to the possibilities of the hospital, the various manipulations performed in the laboratory with a *Staphylococcus epidermidis* demonstrate the necessity to realize the culturing of intraoperative specimens as quickly as possible, considering the decay of growth opportunities when delay before culturing exceeds 2 hours and the subsequent risk of false negative. The use of temporary storage at 4°C has a positive influence on the conservation of bacterial strains, mainly during the first 4h. In the study of Trampuz et al, samples obtained after the sonicated prostheses were supported by bacteriologists within 6h [10]. This element could probably explain part of the improved sensitivity of the sonication compared to the simple cultivation of intraoperative biopsies, even if the original purpose of the sonication is rather to release the bacterial strains in the buried biofilm [11].

A limitation of this study was that only a single aerobic strain was cultured and, as a consequence the conclusions can’t be extended to all microorganisms causing PJI. Nevertheless, it helps in determining the best method of preservation and transmission of microbiological samples to optimally isolate and identify the infectious germ. It would be interesting to carry out the same study with others microorganisms. Currently, we don’t realize the study on other strains because the necessary osseous stock is considerable and impacts on our tissues Bank.

The virulence (sensitivity) of the crop is also influenced by the clinical setting. Font-Vizcarra reported that the synovial fluid, tissue around the implant and even swabs, were more frequently positive in acute infections than in chronic infections (96% vs. 82% for the liquid, 87% versus 74% for the biopsy, and 87% versus 44% for the swab) [12]. Possibly, acute infections include more virulent germs with higher growth velocity and higher inoculum. As the biofilm is not formed yet, the bacterium is in active form [13].

Bjerkan et Al. also demonstrated that the site of the biopsies could alter the infection rate; samples in contact with the implant in the interfaces, resulted in more positive microbiological results than biopsies taken from the capsule, and even analysis of synovial fluid [14]. The timing at which biopsies are analyzed also seems to play a key role: late treatment of biopsies, stored in microbiological standard jars, could fail to identify fragile or strict anaerobic germs. This may explain their low presence in epidemiology of prosthetic infections.

In our protocol, bone fragments were obtained from patients who received prophylactic antibiotics at induction. This treatment didn’t manage to completely inhibit the growth of bone-infecting germs, but the autoclave of the bone, which could be seen as an eradication of the antibiotics, allowed a better growth of the bacterial strain (Fig 5). The effect of autoclave could be twofold; *i*. On the proteolytic degradation of the antibody and therefore a decrease in the immune activity of the host bacteria or *ii*. Degrade the antibiotic molecules still present in the sample. Cefazolin remains active at low temperatures and as such, could participate in the relative inhibition of the bacterial culture. Burnett et al demonstrate that prophylactic antibiotics do not affect cultures during prosthetic surgery [15]. Nevertheless, our study was not designed to demonstrate this, since antibiotic prophylaxis reach a peak concentration 30 min post injection and is already fading away by the third hour. This is also the reason why the injection of a second dose might be required should the surgery need to be prolonged.

While the extent of the antibiotics treatment is still at question, some authors recommend its upholding until new samples are taken [16]. In that regard, Zimmerli *et al*. suggest a therapeutic window of 15 days between 2 sampling. This time window should allow the microbiological sterility of the site to be confirmed before reconstruction [5], [6]. The therapeutic window may be influenced by the extensive tissue bioavailability of certain antibiotics such as quinolones or rifampicin, justifying our therapeutic experience window of approximately a month. This latency is also preferred when placing a support for sustained local antibiotic release [17–20]. Some authors consider the inhibition of culture to be even longer and therefore justify the use of other diagnostic methods such as PCR or sonication or both. Both techniques indeed seem less influenced by previous antibiotic therapies [11], [13]. However, these techniques require many handling steps and would be more likely to generate false positives and, as a result, decrease the specificity [10], [11], [13], [21]. The false positive rate is much lower in this research compared to other studies (4% *vs* up to 13.4%, respectively)[22], [23]. It is likely that this rate could be reduced if the seeding were done near an orthopedic operating room, rather than in a laboratory (sterile field, sterile gown, and sterile gloves), provided that the Technical seeding is acquired by the surgical team and appropriate culture media are used. However this is not always possible in all Hospital especially small hospital with less infrastructures.

## Conclusion

The identification of the germ responsible for prosthetic infection is a key element in the management of the chronic prosthetic joint infections. Due to the biofilm, the bacterial growth is inversely proportional to the delay before culture. Concerning *the Staphylococcus epidermidis*, our study shows the need to cultivate as soon as possible and optimally within 2h after the completion of the sampling. Temporary storage in a refrigerator at 4°C also appears to have a positive influence on bacterial viability. At present, these conclusions concern only the Staphylococcus epidermidis and must be tested on the other bacteria to be validated. To ensure these favorable conditions, a timely communication with the microbiologist is key and cultivation directly in the operating room is recommended, if possible. Further investigation is required and could demonstrate the superiority of this method over current seeding procedures in the laboratory.

## Supporting information

S1 Table Data. (XLSX)
